# Standardized Methodology of Scaffold-Free and Scaffold-Based 3D Epithelial Spheroid Culture for Skin Regenerative Research

**DOI:** 10.3390/mps8050123

**Published:** 2025-10-16

**Authors:** Mariana B. Ramos-Pinto, Maria Leticia de Almeida Lança, Cristiane H. Squarize, Rogerio M. Castilho

**Affiliations:** 1 Laboratory of Epithelial Biology, Department of Periodontics and Oral Medicine, School of Dentistry, University of Michigan, Ann Arbor, MI 48104, USA; marianp@umich.edu (M.B.R.-P.);; 2 Department of Bioscience and Oral Diagnosis, Institute of Science and Technology, São Paulo State University—UNESP, São José dos Campos 12245-000, SP, Brazil; 3 Rogel Cancer Center, University of Michigan, Ann Arbor, MI 48109, USA

**Keywords:** epithelial spheroids, ultra-low-attachment culture, high-throughput screening, matrigel scaffold, ROCK1 inhibition

## Abstract

*Background:* Extensive skin injuries from severe burns or chronic non-healing ulcers overwhelm the body’s natural repair mechanisms, while current therapeutic approaches relying on autologous skin grafting are limited by donor site availability. Three-dimensional epithelial spheroid cultures enhance stem cell regenerative potential, but standardized comparative methodologies are lacking. *Methods*: We established a comprehensive framework comparing scaffold-free and scaffold-based epithelial spheroid systems using HaCaT keratinocytes. High-throughput approaches utilized BioFloat and ELPLASIA 96-well platforms, while low-throughput 6-well ULA plates generated heterogeneous populations (holospheres, merospheres, paraspheres). Scaffold-based studies embedded spheroids in Matrigel to evaluate outgrowth capacity. ROCK1 inhibitor treatment was assessed for stemness enhancement. *Results:* High-throughput systems generated uniform spheroids with high reproducibility and consistent circularity. Low-throughput cultures produced heterogeneous populations with distinct size distributions (holospheres: 408.7 μm^2^, merospheres: 99 μm^2^, paraspheres: 14.1 μm^2^). In Matrigel scaffolds, merospheres and paraspheres migrated outward, forming epithelial sheets, while holospheres remained intact as BMI-1+ stem cell reservoirs. ROCK1 inhibition enhanced holosphere formation, preserved stemness markers, and reduced premature differentiation. *Conclusions:* This standardized toolbox demonstrates scaffold-free systems optimize scalability for screening while scaffold-based approaches enable physiologically relevant regenerative studies. Integration of both methodologies provides flexibility matching experimental design to scientific objectives, accelerating translation to clinical applications.

## 1. Introduction

The skin has a remarkable capacity for rapid reepithelialization, and barrier restoration is central to wound healing; however, in extensive injuries, this natural process is often overwhelmed, leading to chronic wounds and significant morbidity. Large-area epithelial defects, whether arising from traumatic accidents, diabetic ulcers, or severe burns, frequently outstrip the body’s intrinsic regenerative potential, necessitating advanced therapeutic interventions [1,2]. Conventional autologous skin grafting, while effective for smaller lesions, is severely limited by donor site availability and patient comorbidities [3]. These challenges underscore the urgent need to develop novel strategies that can reliably deliver functional skin tissue or skin-forming cells to patients with extensive epithelial loss, thereby improving clinical outcomes and reducing the burden of prolonged hospital stays.

Severe burns are life-threatening conditions that, unless managed properly, can lead to severe systemic impact, fast organ failure, and death. Death is mainly driven by the size and depth of the burn [4]. The major prevalence of burns occurs due to exposure to flames in young children and the adult population between 20 and 55 years old [5]. Sepsis is reported in one-quarter of all adult burn patients, with a mortality rate of about 34% [6]. Patients with large burn areas are initially treated with specialized bandages and dressings to protect the wound bed and prevent infection. Still, the inherently slow pace of re-epithelialization prolongs open-wound exposure, heightening the risk of microbial invasion and septic complications [7]. For wounds covering more than 60% of the total body surface area, treatment often entails a combination of allografts from skin banks, the application of dermal substitutes, and repeated autologous graft harvests every 2–3 weeks [8]. Laboratory-grown skin constructs have been employed for decades, yet mixed clinical outcomes persist, largely because extended culture times limit the timely availability of graftable tissue [9,10,11]. These limitations highlight the pressing need for new therapeutic approaches that accelerate wound closure by enhancing the stemness potential of epithelial cells prior to grafting.

In recent years, three-dimensional culture systems have emerged as a powerful means to boost epithelial cell stemness in vitro, with low-adhesion spheroid cultures at the forefront of these innovations [12]. By culturing epithelial cells under non-adherent or low-attachment conditions, cells spontaneously aggregate into spheroidal microtissues that recapitulate key aspects of stem cells, including enhanced expression of stem cell markers. Within these spheroids, enhanced cell–cell and cell–matrix interactions foster the upregulation of progenitor markers, improved proliferative capacity, and greater resistance to apoptosis [13,14,15]. Multiple spheroid culture modalities have been adapted for epithelial biology, including scaffold-based hydrogels that mimic extracellular matrix cues and fully scaffold-free systems that permit high-density, scalable expansion. Collectively, these approaches hold great promise for generating epithelial populations with superior regenerative potential.

Here, we present a comparative study of distinct methodological strategies for culturing epithelial cell spheroids tailored to diverse research and translational objectives. The primary innovation of this study lies in establishing a comprehensive, integrated methodological framework that systematically optimizes three-dimensional spheroid culture systems for epithelial regenerative applications. While individual spheroid culture techniques exist, the field lacks standardized, comparative methodological protocols that enable researchers to select the most appropriate approach for their specific experimental objectives. Our innovation addresses this critical gap by presenting a unified strategy that encompasses three distinct approaches. First, we describe scaffold-free culture strategies optimized for high-throughput, homogeneous spheroid generation, suitable for applications requiring bulk cell expansion, ranging from small molecule drug screening to CRISPR-based library screens. Second, we outline low-throughput techniques that yield heterogeneous spheroid populations, enabling in-depth interrogation of epithelial stem cell subpopulations and their unique stemness characteristics. Finally, we detail low-throughput strategies that emphasize the study of epithelial stem cell heterogeneity using scaffold-incorporated approaches, which explore the behavior of spheroids growing inside reconstituted basal membrane matrices. This comprehensive methodological advancement provides the research community with a robust, reproducible toolbox for epithelial stem cell cultivation, ultimately accelerating the translation of spheroid-based therapies from laboratory bench to clinical applications and equipping researchers with the flexibility to select the optimal spheroid culture platform for their specific goals, whether clinical-scale tissue engineering or fundamental studies of epithelial progenitor biology.

## 2. Material and Methods

### 2.1. Cell Line and Routine Culture

The human keratinocyte line HaCaT used in our study is a well-established, immortalized human keratinocyte cell line that was originally developed and characterized by Boukamp et al. [16]. The cells were maintained in Dulbecco’s Modified Eagle’s Medium (DMEM; Gibco, Cat. No. 11965-092) supplemented with 10% heat-inactivated fetal bovine serum (FBS; Gibco, Cat. No. 26140-079), 100 U/mL penicillin, 100 µg/mL streptomycin (Gibco, Cat. No. 15140-122), and 250 ng/mL amphotericin B (Gibco, Cat. No. 15290-018). Cells were passaged at 70–80% confluence using 0.05% trypsin-EDTA (Gibco, Cat. No. 25300-054) and reseeded at a 1:5 split ratio. All cultures were maintained in a humidified incubator at 37 °C, 5% CO_2_, and routinely tested negative for mycoplasma contamination (MycoAlert, Lonza). In this study, we use the terms low-throughput and high-throughput to distinguish between different spheroid culture formats. Low-throughput models used six-well ultra-low-attachment (ULA) plates, which produce hundreds of spheroids per well with a wide range of sizes and shapes, making them ideal for studying stemness diversity and biological behavior at the population level. In contrast, high-throughput models (96-well BIOFLOAT and Elplasia plates) generate large numbers of uniform spheroids in parallel, offering scalability, reproducibility, and compatibility with automated imaging pipelines. Together, these complementary approaches allow both detailed exploration of epithelial heterogeneity and efficient, standardized production of spheroids for screening or translational applications.

### 2.2. High-Throughput Spheroid Formation

Two commercial ULA platforms were used in parallel for high-throughput spheroid generation. Prior to cell seeding, plates were pre-incubated with complete DMEM for 30 min at 37 °C to equilibrate temperature.

- Elplasia 96-well Black Round Bottom Microcavity plate (Corning, Cat. No. 4442): HaCaT cells were trypsinized, counted by trypan blue exclusion (Countess II, Thermo Fisher), and resuspended at 1.0 × 10^6^ cells/mL. A 50 µL aliquot (5.0 × 10^4^ cells) was gently dispensed into each well to populate the microcavities. Plates were incubated undisturbed for 48 h at 37 °C, 5% CO_2_.
- BIOFLOAT 96-well U-Bottom plate (Sarstedt, Cat. No. 83.3925.400): Cells were resuspended at 1.0 × 10^5^ cells/mL, and 50 µL (5.0 × 10^3^ cells) was added per well. Plates were incubated undisturbed for 48 h at 37 °C, 5% CO_2_.

On day 2, four non-overlapping fields per well were imaged at 4× magnification using the ImageXpress Micro 4 (Molecular Devices, LLC., Sao Jose, CA, USA) in phase-contrast mode. Automated analysis (MetaXpress® High-Content Image Acquisition and Analysis Software, v.6) quantified spheroid number, diameter, and circularity using a standard thresholding algorithm (minimum object size 50 µm^2^, circularity > 0.6). Triplicate plates were analyzed per platform.

### 2.3. Low-Throughput Spheroid Heterogeneity Assay

To investigate spheroid subtype formation under low-throughput conditions, 8.0 × 10^3^ HaCaT cells in 2 mL complete DMEM were seeded into each well of ULA six-well plates (Corning, Cat. No. 3471) in sextuplicate. We selected the six-well ULA format for these assays because it promotes the formation of a heterogeneous population of spheroids of varying sizes and morphologies, allowing the emergence of holospheres, merospheres, and parashperes that reflect different proliferative and stem-like potentials. Moreover, spheroids generated in six-well cultures are larger and more structurally robust, which facilitates downstream handling, embedding in Matrigel, and quantitative imaging analyses. Two experimental groups were established: control and Rho-Kinase (ROCK1) inhibitor (Y-27632; Tocris, Cat. No. 1254) at 5 µM final concentration (diluted from 10 mM stock solution in DMSO). Plates were incubated for five days without medium change. On day 5, spheroid morphology was assessed using an inverted EVOS Cell Imaging System (Thermo Fisher) under brightfield optics (20× objective). Spheroids were classified by two independent observers according to size and morphology, consistent with previous literature. To standardize classification, spheroid cross-sectional areas were quantified in ImageJ and stratified into quartiles. Holospheres were defined as large, smooth, compact spheroids corresponding to the upper quartile of sizes (>200 µm); merospheres as intermediate spheroids within the interquartile range (75–200 µm); and parashperes as small, irregular spheroids in the lowest quartile (<75 µm). Approximately 200 spheroids per well were counted, and subtype frequencies were recorded.

### 2.4. Scaffold-Based Outgrowth and Immunofluorescence

On day 5 of ULA culture, spheroids from each group were pooled by gentle pipetting, collected into 15 mL tubes, and centrifuged at 200× *g* for 5 min at room temperature. Supernatant was removed, and spheroid pellets were gently resuspended in ice-cold, growth factor-reduced Matrigel (Corning, Cat. No. 356231) at a concentration of ~50 spheroids per 50 µL matrix. Fifty microliters of the spheroid–Matrigel suspension were dispensed into each well of pre-warmed 24-well plates (Greiner Bio-One, Cat. No. 662160), allowed to polymerize for 30 min at 37 °C, and overlaid with 500 µL complete medium ±5 µM Y-27632. Cultures were maintained for seven days with medium replacement every other day.

At day 7, scaffolds were fixed with 4% paraformaldehyde (Electron Microscopy Sciences, Cat. No. 15710) for 20 min at room temperature, then washed three times with 1× PBS (Gibco) for 5 min each. Permeabilization and blocking were performed in PBS containing 1% bovine serum albumin (BSA; Sigma-Aldrich, Cat. No. A9418) and 0.5% Triton X-100 (Sigma-Aldrich, Cat. No. T8787) for 60 min at room temperature with gentle rocking. Samples were then incubated overnight at 4 °C with primary antibodies diluted in blocking buffer: mouse anti-BMI-1 (1:10; Developmental Studies Hybridoma Bank, Cat. No. Bmi1), rabbit anti-Cytokeratin 10 (CK10; 1:500; Abcam, Cat. No. ab76318), and rabbit anti-Cytokeratin 14 (CK14; 1:200; Santa Cruz Biotechnology, Cat. No. sc-53253). After three PBS washes, scaffolds were incubated for 1 h at room temperature in the dark with Alexa Fluor 488-conjugated goat anti-mouse IgG and Alexa Fluor 568-conjugated goat anti-rabbit IgG (1:500; Invitrogen), plus Hoechst 33342 nuclear stain (1:500; Invitrogen, Cat. No. H3570). Final washes were performed in PBS before adding 200 µL fresh PBS to each well.

### 2.5. Image Acquisition and Analysis

For the spheroid outgrowth area quantification in the scaffold-based model, entire wells were imaged using an inverted EVOS Cell Imaging System (Thermo Fisher) under brightfield optics (4× objective). The outgrowth was defined as the total area beyond the spheroid core segmented in ImageJ. For the immunofluorescence analysis of the scaffold-based model, entire wells were imaged at 10× magnification on the ImageXpress Micro 4 (Molecular Devices, LLC., Sao Jose, CA, USA) using Hoechst, FITC, and Texas Red filter sets. For each well, four corner fields and the center field were acquired as z-stacks (3 planes, 10 µm step), and maximum-intensity projections were generated. Fluorescence quantification was performed using the MetaXpress® High-Content Image Acquisition and Analysis Software, v.6. Thresholds were set at the mean background plus 2 standard deviations, and the total positive area per channel was measured. Data represent mean ± SEM from six biological replicates per condition.

### 2.6. Statistical Analysis

All experiments were conducted in at least three independent biological replicates. Quantitative data were analyzed using GraphPad Prism 10.0. The Shapiro–Wilk test was used to assess data normality when possible. If the result was a parametric distribution, a one-way ANOVA followed by a Tukey post hoc analysis was applied to multiple group comparisons. For two-group comparisons, an unpaired two-tailed Student’s *t*-test was employed. For nonparametric distributions, comparisons between two groups used the unpaired two-tailed Mann–Whitney test. Asterisks denote statistical significance, with *p*-values reported as follows: * *p* < 0.05, ** *p* < 0.01, *** *p* < 0.001, **** *p* < 0.0001.

### 2.7. Volumetric Spheroid Analysis and Circularity Measurements

To quantitatively assess spheroid shape and volume, we acquired 12 z-stack images of live spheroids on day 2 (high-throughput assays) or day 5 (low-throughput assays) using the ImageXpress Micro 4 in bright-field mode (10× objective) with 12 optical sections at 10 µm intervals. For each well, a central field was selected and exported as a multi-plane TIFF. All image processing and analysis were performed in ImageJ (v1.54g). First, each z-stack was converted to an 8-bit image and background-subtracted using a rolling ball radius of 50 pixels. Spheroid boundaries were enhanced by applying a median filter (radius = 2 pixels) and then thresholded to create a binary mask. To count the spheres and calculate the sphere width (µm), we used freehand lines to measure and record the results for each spheroid. To measure circularity, we generated maximum-intensity projections of the same z-stack and ran “Analyze > Analyze Particles” with size 100–∞ µm^2^ and circularity 0.00–1.00. Circularity was calculated by ImageJ as Circularity = 4π × (Area)/(Perimeter^2^), where a value of 1.0 indicates a perfect circle (https://imagej.net/ij/docs/menus/analyze.html). For each condition, all the spheroids included in the image were analyzed across three biological replicates. Volumetric and circularity data were exported to GraphPad Prism for statistical analysis as described above.

## 3. Results

In the current study, we evaluated and compared high-throughput, scaffold-free methodological strategies for culturing and expanding epithelial cell spheroids.

### 3.1. Scaffold-Free High-Throughput Strategies to Culture Epithelial Spheroids

**Figure 1:**
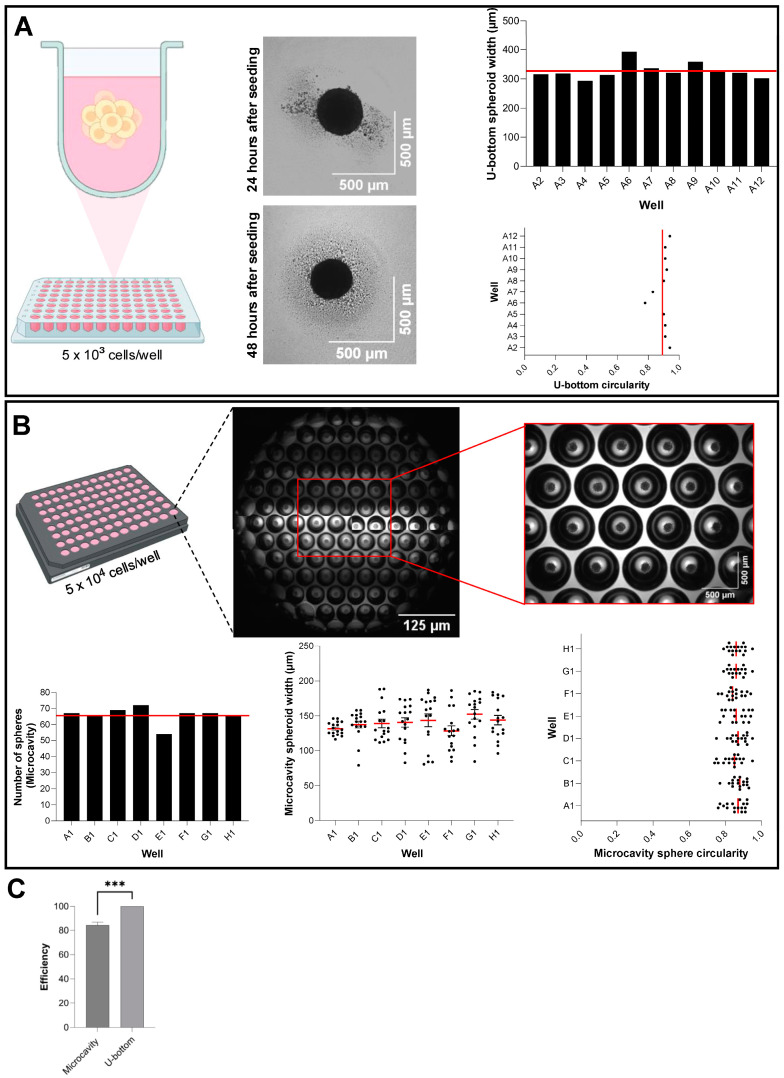
Scaffold-free high-throughput culture of epithelial spheroids. (**A**) Diagram representing sphere formation in each well of the 96-well plate (left panel), brightfield representative images of spheroids captured after 48 h in the BIOFLOAT U-Bottom plate (center panel). Quantitative analysis of 12 wells showing size uniformity (325.21 μm ± 99) and consistent circularity values (right panel). (**B**) Diagram representing sphere formation in Elplasia 96-well Round Bottom Microcavity plate containing 79 microcavities (Top panel). Efficiency of sphere-forming per well along spheroid width and circularity is shown (lower panel). (**C**) Comparative analysis of spheroid-forming efficiency between BIOFLOAT U-Bottom plate and Elplasia 96-well Round Bottom Microcavity plate is shown. Data represent mean ± SEM of independent biological replicates (8 replicates for the number of spheres, and 17 replicates for width and circularity). Statistical comparison as performed using unpaired Mann–Whitney test for spheroid-forming efficiency (*p* = 0.0002). Asterisks indicate significance levels: *** *p* < 0.001.

To generate three-dimensional epithelial aggregates, we employed low-adhesion culture approaches using two commercially available ULA platforms: the BIOFLOAT U-Bottom plate (Sarstedt) and the Elplasia 96-well Round Bottom Microcavity plate (Corning). The BIOFLOAT system features an anti-adhesive polymer coating that modifies the plastic surface of each well, creating an exceptionally uniform, highly anti-adhesive surface. This allows adherent cells to preferentially form cell–cell contacts and assemble into three-dimensional spheroids without adhering to the vessel walls. In our experiments, epithelial cells seeded into BIOFLOAT U-Bottom wells formed one well-rounded spheroid after 48 h (Figure 1A diagram and bright field images of spheroids). Quantitative analysis confirmed robust size uniformity across wells (Figure 1A middle panel—325.21 μm ± 99) and high circularity values ranging from 0.78 to 0.94 (unitless), averaging after 48 h of culture (Figure 1A right panel). Next, we evaluated the Elplasia Microcavity plate, which contains microcavities in a standard 96-well format, allowing a theoretical yield of 7584 spheroids per plate (79 spheroids/well) (Figure 1B top panel). When seeding 5 × 10^4^ cells per well, we consistently obtained an average of 68 individual spheroids per well (Figure 1B lower left), translating to approximately 6528 spheroids per 96-well plate. Spheroids formed in the Elplasia system exhibited a narrow size distribution with an average width of 150 µm and circularity values ranging from 0.74 to 0.95, with a mean of 0.85 (unitless) (Figure 1B lower right graphics).

A direct comparison of both platforms revealed that the BIOFLOAT U-Bottom system achieved nearly 100% spheroid-forming efficiency (one spheroid per well), whereas the Elplasia microcavity system reached an 84.2% efficiency per well (*** *p* < 0.0001) (Figure 1C). Despite this difference in efficiency, the microcavity format still produced a high absolute number of spheroids per well, and due to the microcavity design, it allows for the change in culture medium and, therefore, prolonged maintenance of spheroids in culture when compared to the U-Bottom system (Supplementary Video S1). Although different plating densities may further influence spheroid yield, we used a standardized seeding number across all platforms to enable direct comparison. Together, these data demonstrate that both scaffold-free systems offer high spheroid-forming capabilities, and the choice between them should be guided by specific experimental throughput and scale requirements.

### 3.2. Exploring the Heterogeneity of Spheroids Using ULA Strategy

Epithelial cells cultured under ultra-low adhesion (ULA) conditions self-organize into three principal spheroid subtypes, holospheres, merospheres, and paraspheres, each reflecting progressively reduced clonogenic and stem-like potential [17]. Holospheres are large, tightly packed spheroids with high proliferative capacity and long-term self-renewal; merospheres exhibit intermediate size and growth characteristics; and paraspheres form small, loosely organized aggregates with limited expansion potential. To dissect the stemness properties of each subtype and capture intrapopulation heterogeneity, a scaffold-free ULA culture plate provides an ideal platform.

**Figure 2:**
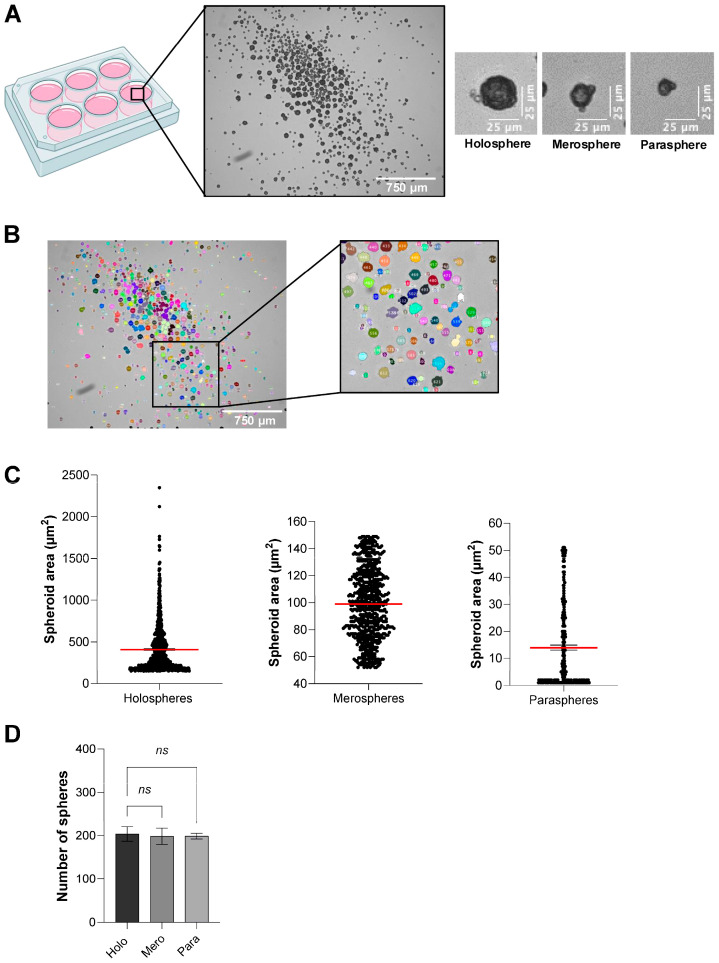
Heterogeneity of epithelial cell stemness using ULA 6-well plate. (**A**) Brightfield micrographs depict mixed populations of holospheres, merospheres, and paraspheres after 48 h of culture. (Scale bar: 750 µm). (**B**) Segmentation of spheroid size using ImageJ (v1.54g, “Color threshold segmentation” and “Analyze Particles”) (Scale bar: 750 µm). (**C**) Quantification of spheroid area distributed within holospheres, merospheres, and paraspheres. (**D**) Quantification of the total number of spheroids classified by its subtypes (holospheres, merospheres, and paraspheres). Data represent mean ± SEM of three independent biological replicates. Statistical comparison as performed using one-way ANOVA for comparing the number of spheres (Holosphere versus Merosphere—*p* = 0.955; Holosphere versus Parasphere—*p* = 0.960) ns *p* > 0.05.

Using ULA six-well plates, we seeded 8 × 10^3^ epithelial cells per well and maintained the cultures without disturbance for 48 h. Under these conditions, a mixed population of holospheres, merospheres, and paraspheres emerged spontaneously (Figure 2A). This heterogeneity in spheroid morphology and size was quantified in ImageJ (v1.54g) using the “color threshold” for segmentation followed by the built-in “Analyze Particles” function (Figure 2B).

Quantitative measurements revealed distinct size distributions for each spheroid class (Figure 2C): holospheres displayed a mean cross-sectional area of 408.7 µm^2^, merospheres averaged 99 µm^2^, and paraspheres averaged 14.1 µm^2^. Furthermore, seeding 8 × 10^3^ cells consistently yielded roughly 200 spheroids of each subtype per well (Figure 2D).

### 3.3. Scaffold-Based Culture of Spheroids

**Figure 3:**
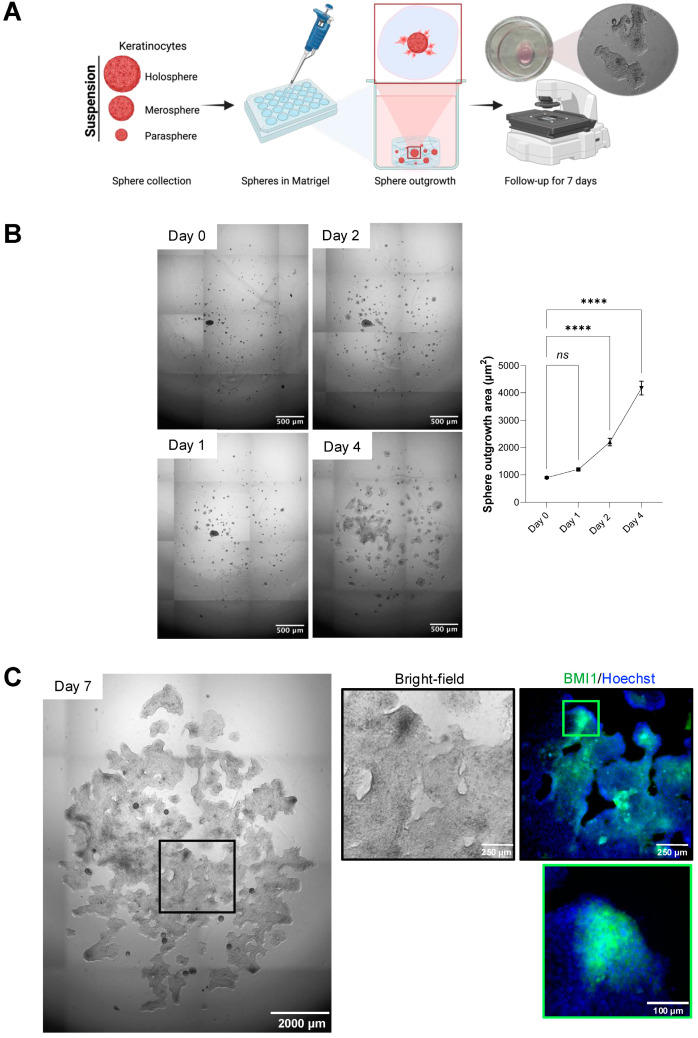
Scaffold-based culture of spheroids. (**A**) Schematic representation of experiment outline demonstrating isolation of spheroids (holospheres, merospheres, and paraspheres) from ULA cultures, mixed with cold Corning Matrigel, cast into wells, and allowed to gel at 37 °C before seven days of culture. (**B**) Brightfield time-course of individual spheroid outgrowth for up to 4 days. Quantification of total outgrowth area. Outgrowth area from 899.12 ± 116 on day 1 to 4174.45 ± 146 on day 4. (Scale bar: 500 µm). (**C**) Brightfield image of spheroid outgrowth by day 7 (left panel) (Scale bar: 2000 µm). High-magnification brightfield and immunofluorescence images (right panel) depict sheets of epithelial cells growing in the matrix, and the presence of BMI-1 positive cells (green) in holosphere cores and surrounding tissue, counterstained with Hoechst (blue) (Scale bar: 250 µm). Higher magnification of spheroid BMI-1+ areas (scale bar: 100 µm). Data represent mean ± SEM of three independent biological replicates. Statistical comparison as performed using one-way ANOVA for comparing the sphere outgrowth area in the control group over time, using day 0 as baseline (Asterisks indicate significance levels: Day 1—ns *p* > 0.05, Day 2 and 4—**** *p* < 0.0001).

To evaluate the capacity of heterogeneous spheroid populations to generate continuous epithelial sheets, an important step for expanding cell numbers to cover large skin defects, we next embedded spheroids in a Matrigel-based scaffold. This assay models how mixed holosphere, merosphere, and parasphere populations might contribute to sheet formation in tissue-engineering applications where rapid epithelial expansion is critical. Figure 3A schematically illustrates the workflow. After 48 h in ULA culture, spheroids were collected (Supplementary Video S2), pooled, and gently mixed with cold Corning Matrigel matrix, a reconstituted basement membrane extracted from EHS mouse tumors that contains laminin, collagen IV, entactin, heparan sulfate proteoglycan, and native growth factors. At 4 °C Matrigel remains liquid for easy handling; raising the temperature to 22–37 °C induces gelation through polymerization of these ECM proteins, creating a supportive three-dimensional environment. The spheroid–Matrigel suspension was cast into culture wells, allowed to gel at 37 °C, and maintained for up to seven days with medium changes every other day.

In Figure 3B, brightfield images show spheroid behavior over time. On day 1, distinct holosphere, merosphere, and parasphere cores are visible within the gel. By day 3, cells begin migrating outward from merospheres and paraspheres to form a planar epithelial sheet. By day 7, this outgrowth has expanded substantially, while holosphere cores remain compact spheres. Quantitative analysis in ImageJ (v1.54g, “Color Threshold” segmentation plus “Analyze Particles”) reveals the total area covered by spheroids plus outgrowth area (µm^2^) increases from 899.12 ± 116 on day 1 to 4174.45 ± 146 on day 4 (ns *p* > 0.05, **** *p* < 0.0001) (Figure 3B). High-magnification immunofluorescence confirms that by day 7, only holosphere-derived spheroid cores retain expression of the stem-cell marker BMI-1, whereas the surrounding epithelial sheet, originating from merospheres and paraspheres, is largely BMI-1 negative (Figure 3C). These results demonstrate that Matrigel embedding both preserves highly stem-like holospheres and drives the outgrowth of less potent spheroids into epithelial sheets, offering a scalable approach for generating large numbers of epithelial cells for skin tissue regeneration.

### 3.4. Epithelial Differentiation Within Matrigel Outgrowth

To determine whether spheroid-derived outgrowth cells undergo terminal differentiation or retain their proliferative capacity, we stained Matrigel-embedded cultures at day 7 for cytokeratin 14 (CK14) and cytokeratin 10 (CK10). CK14 is a canonical marker of basal epidermal keratinocytes that retain proliferative capacity and, in native skin, is confined to the basal layer. In contrast, CK10 marks spinous-layer cells that have initiated terminal differentiation and no longer proliferate.

**Figure 4:**
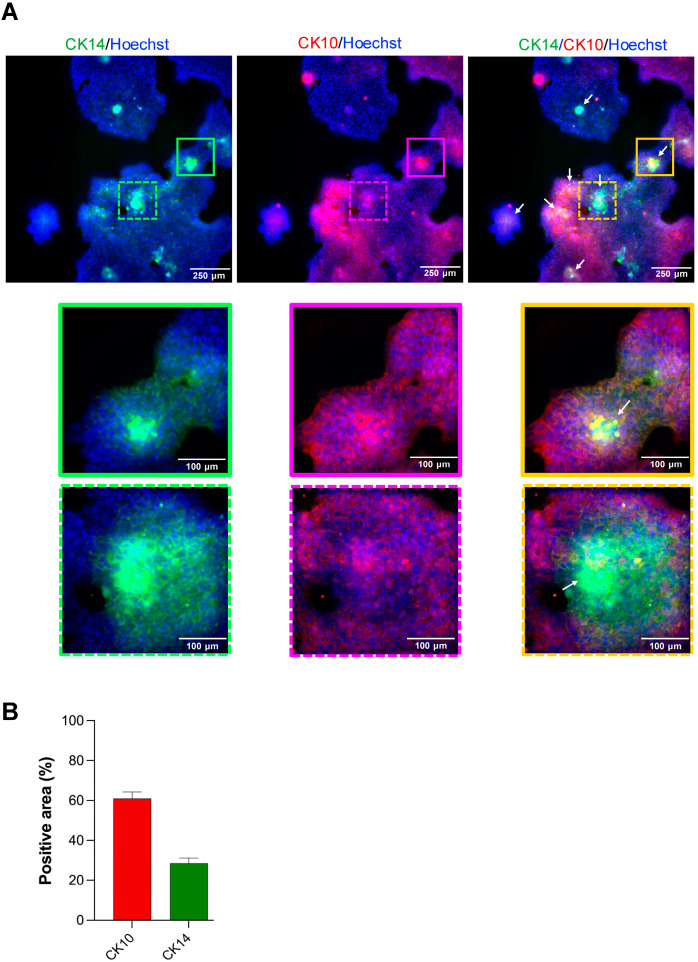
Cellular differentiation of spheroids outgrowth in Matrigel. (**A**) Confocal images show cytokeratin 14 (CK14—green) and cytokeratin 10 (CK10—red) positive cells and counterstained for DNA content using Hoechst (blue) (Scale bar: 250 µm) (Top panel). Insert magnification of the spheroid outgrowth into the matrix (Scale bar: 100 µm) (Lower panel). (**B**) Quantification of total positive area for CK14 and CK10 within the Matrigel. Note concentration of CK14-positive cells within the spheroids (arrows) (CK10-positive outgrowth area 61 ± 7.9%, CK14-positive outgrowth area 28.8 ± 6.2% remains, mean ± SEM, *n* = 12 fields).

In Figure 4A, confocal images show a robust CK14 (green) signal restricted to the holospheres distributed throughout the Matrigel. Surrounding these spheroids, cells migrating into the matrix (outgrowth area) express CK10 (red), indicating they have exited the basal-progenitor state and entered the differentiation program. Nuclei are counterstained with Hoechst (blue). Quantitative image analysis (ImageJ v1.54g “Analyze Particles” following threshold segmentation) of twelve independent fields revealed that the outgrowth population is predominantly CK10-positive. As summarized in Figure 4B, CK10+ area accounts for approximately 61 ± 7.9% of the total epithelial outgrowth, whereas CK14+ signal outside of the spheroid cores comprises only 28.8 ± 6.2%. These data confirm that merosphere- and parasphere-derived cells migrating from the spheroids primarily adopt a differentiated spinous-layer phenotype, while the holosphere spheroids maintain a basal-like, proliferative identity.

### 3.5. Pharmacological Enhancement of Stemness Potential

Mammalian cell stemness can be modulated by genetic reprogramming or chemical means. For example, overexpression of the Yamanaka factors (Oct3/4, Sox2, Klf4, c-Myc), which are highly expressed in embryonic stem cells, can induce pluripotency in mouse and human somatic cells by engaging key developmental signaling networks. In parallel, small-molecule inhibitors such as the ROCK1 inhibitor (Y-27632) are widely used to improve survival, prevent apoptosis, and maintain stem-cell–like phenotypes in vitro [1,18,19,20,21]. Here, we tested the effects of supplementing the culture medium of epithelial spheroids grown under ULA conditions with ROCK1 inhibitor, which could further enhance their stemness potential.

**Figure 5:**
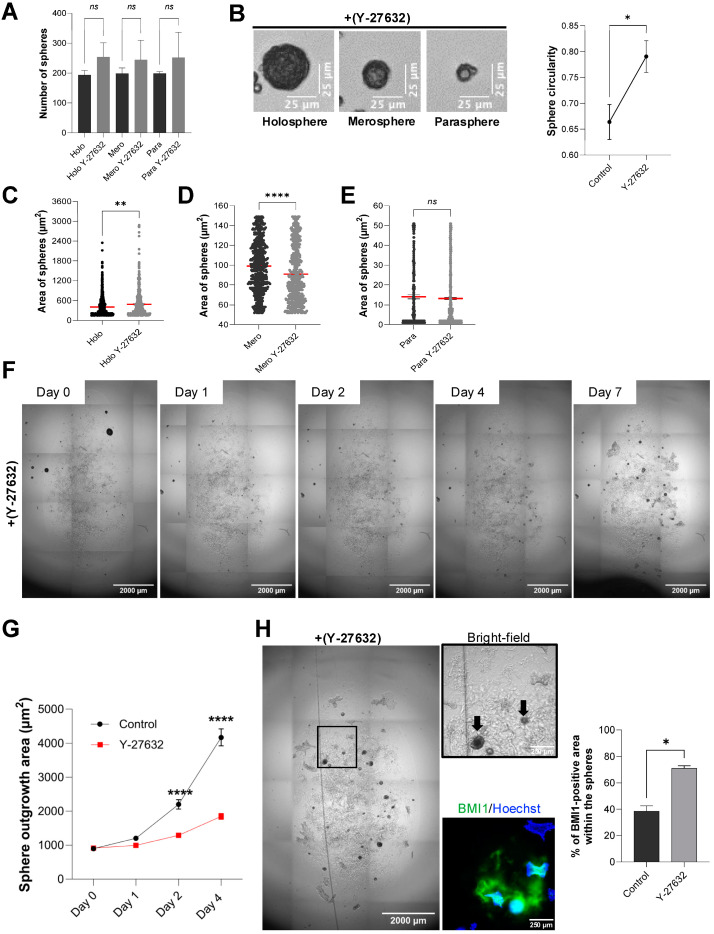
Rock-1-induced enhanced stemness. (**A**) Quantification of ULA spheroids distributed as holospheres, merospheres, and paraspheres receiving Y-27632 or vehicle (48 h) (*n* = all the spheres in 3 wells/group) (ns *p* > 0.05). (**B**) Representative brightfield images of ULA spheroids receiving Y-27632 for 48 h. Graphical representation of enhanced circularity of spheroids receiving Y-27632 compared with vehicle controls (Scale bar: 25 µm) (*n* = all the spheres in 3 wells/group, unpaired Student’s *t*-test * *p* < 0.05). (**C**–**E**) Measurement of spheroid area distributed in holospheres, merospheres, and paraspheres receiving Y-27632 or vehicle (48 h) (ns *p* > 0.05, ** *p* < 0.01, **** *p* < 0.0001) (*n* = all the spheres in 3 wells/group). (**F**,**G**) Spheroids embedded in Matrigel receiving Y-27632 or vehicle and cultured for 7 days. Note the expansion of the holosphere size when receiving Y-27632 and reduced cellular outgrowth (Scale bar: 2000 µm) (*n* = 40 spheres per group per day, two-way ANOVA for comparison between the days and groups statistical significance on days 2 and 4 **** *p* < 0.0001). (**H**) Brightfield images of Y-27632 spheroids growing in Matrigel for 7 days, receiving Y-27632 (Scale bar: 2000 µm) (left panel). Higher magnification brightfield and immunofluorescence images (right panel) of constructs depicts the localization of spheroids within the matrix (arrows), and the presence of BMI-1 positive cells (green) in holospheres, counterstained with Hoechst (blue) (Scale bar: 250 µm). Graphical representation of total BMI-1 positive area within each spheroid and receiving Y-27632 or vehicle (Scale bar: 250 µm) (unpaired Student’s *t*-test * *p* < 0.05).

When ULA spheroids were cultured for 48 h in the presence of 10 µM Y-27632, we observed no change in total number of spheroids compared to controls (ns *p* > 0.05) (Figure 5A). However, morphology assessment revealed that ROCK1 inhibitor treatment produced more dense, well-rounded holospheres, merospheres, and paraspheres and an overall increased spheroid circularity (* *p* < 0.05) (Figure 5B). Quantitative area measurements showed a significant increase in holosphere area size (Figure 5C; ** *p* < 0.01) and the opposite for the merosphere area (**** *p* < 0.0001) (Figure 5D); whereas parasphere area remained unchanged (ns *p* > 0.05) (Figure 5E).

To evaluate whether ROCK1i similarly affects spheroid behavior in a three-dimensional scaffold, we embedded ULA spheroids in Matrigel and maintained them for seven days with continuous ROCK1i supplementation. As shown in Figure 5F,G, treated cultures retained intact spheroids throughout the course and further increased holosphere core size, while the outward migration of epithelial cells was markedly reduced compared to control. Immunostaining for the stem-cell marker BMI-1 confirmed that ROCK1 inhibitor-treated spheroids preserved high BMI-1 expression at day 7 (* *p* < 0.05) (Figure 5H). Interestingly, we could not observe positive cells for cytokeratin 10 on ROCK1i-treated cells.

## 4. Discussion

A deeper understanding of epithelial stem cell biology is fundamental to advancing treatments for patients with extensive wounds, such as severe burns, and those suffering from chronic, non-healing ulcers like diabetic foot lesions. The current gold standard, autologous skin grafting, is critically hampered by the limited availability of donor skin, an issue that becomes acute in patients with large-area injuries [4,22]. Endogenous repair in these settings is insufficient, and repeated harvests from healthy sites can further compromise patient health. Novel cell-based therapies capable of generating large quantities of functional epithelial tissue from a small biopsy are therefore urgently needed. Epithelial spheroids represent a promising strategy: they concentrate cells with high regenerative potential into three-dimensional microtissues that can be directly applied to wounds and, at the same time, serve as a robust platform for high-throughput drug or genetic screening to rapidly identify new agents that enhance skin regeneration.

The methodological approaches presented in this study offer distinct advantages depending on the specific research objectives and experimental requirements. For high-throughput applications where standardization and reproducibility are paramount, we compared two complementary platforms: the BioFloat ultra-low adhesion 96-well plates and the Elplasia system. The BioFloat ultra-low adhesion 96-well plates provide a straightforward approach to generating uniform spheroids through surface modification that prevents cell adhesion while maintaining standard plate dimensions, making them ideal for drug screening applications where spheroid size uniformity is critical for accurate dose–response relationships. In contrast, the Elplasia system represents a more sophisticated approach, featuring 96 wells with each containing 79 individual microwells designed to generate exactly one spheroid per microwell. This innovative design ensures exceptional size uniformity and eliminates the variability often encountered in conventional low-adhesion systems, making it particularly valuable for high-throughput screening applications requiring precise control over spheroid number and size distribution, such as CRISPR-based library screens or compound libraries where consistent spheroid morphology is essential for reliable readouts.

In this study, we compared low-throughput and high-throughput methodologies for generating epithelial spheroids, each tailored to specific experimental objectives. Both high-throughput systems excel in applications demanding large-scale spheroid production with minimal size variation, producing up to thousands of highly uniform spheroids suitable for large-scale screening campaigns, whether testing small-molecule libraries (for example, ROCK1 inhibitors like Y-27632) or performing CRISPR-based genetic screens to uncover new regulators of re-epithelialization [23]. Low-throughput culture in standard six-well ULA plates yields a heterogeneous mixture of holospheres, merospheres, and paraspheres, subtypes that recapitulate different levels of stemness and proliferative capacity [17,24,25]. This size heterogeneity is not a limitation but rather a feature that enables researchers to investigate the relationship between spheroid architecture and stem cell potency, making this approach invaluable for dissecting the intrinsic heterogeneity of epithelial stem cell populations and studying lineage commitment at single-spheroid resolution.

While spheroid size heterogeneity provides insight into intrinsic differences in stem cell potency, the surrounding extracellular matrix adds an additional layer of regulation by providing environmental cues that shape cell behavior. For investigations focused on understanding spheroid heterogeneity and stem cell biology, ULA 6-well plates and scaffold-incorporated culture systems using reconstituted basement membrane matrices, such as Matrigel, provide superior experimental flexibility and add another dimension to spheroid biology studies. When spheroids are cultured within three-dimensional ECM environments, they exhibit altered growth patterns, enhanced cell–matrix interactions, and modified gene expression profiles compared to scaffold-free cultures. The ECM provides biochemical and mechanical cues that influence spheroid development, promoting more physiologically relevant tissue architecture and enabling the study of how extracellular matrix components modulate stem cell behavior and differentiation potential. This scaffold-incorporated approach is particularly valuable for understanding how ECM composition affects spheroid heterogeneity and for investigating the mechanisms underlying stem cell niche interactions, with spheroids grown in matrix-rich environments often displaying enhanced expression of stemness markers and improved engraftment potential. By matching the platform to the question at hand, heterogeneity analysis versus scalable, automated screens, researchers can accelerate both fundamental discoveries and the development of clinically translatable therapies. Although our 96-well ULA platforms enabled the simultaneous generation of thousands of uniform spheroids suitable for imaging and quantitative analysis, we recognize that this system does not constitute a fully automated high-throughput pipeline. Robotic pipetting and automated media exchange would further enhance scalability and reproducibility, but these were beyond the scope of this study. Therefore, our use of the term *high-throughput* refers to the relative increase in spheroid yield and uniformity compared to six-well ULA formats, rather than a fully automated platform.

A critical feature of epithelial spheroids for therapeutic application is their ability to act as regenerative units that facilitate wound coverage through cellular outgrowth. Our scaffold-based model, embedding spheroids in a Matrigel matrix, effectively recapitulates this process. Merosphere- and parasphere-derived cells readily migrate outward to form a continuous epithelial sheet, modeling the initial phase of re-epithelialization. Meanwhile, holosphere spheroids, enriched for stem-like cells expressing BMI-1 and CK14, remain intact and may serve as a stable reservoir poised for long-term renewal. This dynamic interplay suggests a cooperative healing model in which transient amplifying populations drive rapid wound closure, while a reserved pool of true stem cells ensures durable barrier restoration. Importantly, our finding that ROCK1 inhibition preserves the stem cell pool within holospheres and prevents premature differentiation, evidenced by sustained BMI-1 expression and the lack of CK10 expression found in the outgrowth, demonstrates a viable pharmacological “priming” strategy. Our results are consistent with previous studies showing ROCK1i prolongs keratinocyte lifespan and stem-like features [1]. Here, we extend this to spheroid models. Pre-treating spheroids with ROCK1i could maximize the number of regenerative-competent cells delivered to the wound bed, paving the way for “off-the-shelf” cellular therapies that overcome donor-site limitations and restore skin barrier function over large areas.

Ultimately, the selection of spheroid culture methodology should be directly dictated by the specific research questions being addressed. High-throughput standardized approaches using BioFloat or Elplasia systems are optimal when the goal is to screen large compound libraries, perform systematic genetic perturbations, or conduct comparative studies requiring minimal experimental variability, prioritizing reproducibility and scalability over biological complexity. Conversely, when the research focus centers on understanding stem cell biology, investigating spheroid heterogeneity, or studying the effects of microenvironmental cues on cellular behavior, low-throughput approaches using ULA plates or scaffold-incorporated systems provide the necessary experimental flexibility and biological relevance, embracing spheroid diversity as a tool for discovery rather than a source of variability to be minimized. The integration of both standardized and heterogeneous culture approaches within a single methodological framework provides researchers with the flexibility to adapt their experimental design to match their scientific objectives, ensuring that the chosen method enhances rather than constrains the potential for meaningful biological insights.

Although this framework highlights the versatility of different culture platforms, a few considerations temper the generalizability of our conclusions. This study has a few limitations that should be noted. First, our methodological comparison was performed solely using the HaCaT immortalized keratinocyte cell line, which may not fully mimic the behavior of primary human keratinocytes or other epithelial cell types. The process of immortalization can change cellular properties, including proliferation rates, differentiation potential, and response to environmental cues, potentially limiting the generalizability of our findings apply to primary tissue-derived cells. Second, although our high-throughput platforms allow for higher spheroid yields and more uniformity, they are not fully automated systems with robotic handling and media exchange capabilities, which limits their scalability for industrial use. Third, the Elplasia system achieved 84.2% efficiency per well, and while we followed manufacturer recommendations for cell seeding density, optimizing seeding parameters may be necessary when using different cell types than epithelial cells. Despite these limitations, our comprehensive methodological framework offers a solid foundation for future research, enabling these specific issues to be addressed while building on the established protocols.

## 5. Conclusions

This study establishes a standardized methodology for generating and analyzing human epithelial spheroids across complementary scaffold-free and scaffold-based platforms. High-throughput ULA systems such as BIOFLOAT and Elplasia plates reliably produced thousands of uniform spheroids, supporting applications that require scalability, homogeneity, and automated imaging. In contrast, six-well ULA cultures generated heterogeneous spheroid populations (holospheres, merospheres, and parashperes) that provided an effective model to investigate differences in proliferative capacity and differentiation potential. Embedding these heterogeneous spheroids in Matrigel recapitulated wound re-epithelialization, with merospheres and parashperes contributing to epithelial sheet outgrowth while holospheres preserved BMI-1 and CK14 expression, serving as a reservoir of stem-like cells. Furthermore, pharmacological priming with ROCK1 inhibition selectively expanded holospheres and merospheres, maintained stem-like marker expression, and reduced premature differentiation, highlighting a feasible strategy to enhance regenerative potential. Together, these findings underscore the value of combining scaffold-free and scaffold-based approaches: while high-throughput systems enable reproducibility and screening, low-throughput heterogeneous models better capture epithelial biology and regenerative dynamics. By integrating these strategies, we provide a practical toolbox for researchers in regenerative medicine, spanning from mechanistic studies of epithelial heterogeneity to the scalable production of spheroids for translational applications.

## Data Availability

The data presented in this study are available on request from the corresponding author.

## Supplementary Materials

The following supporting information can be downloaded at https://www.mdpi.com/article/10.3390/mps8050123/s1. Supplementary Video S1: Gentle media exchange in Elplasia microcavity wells containing spheroids. The video shows careful aspiration of spent medium followed by slow addition of fresh DMEM via pipette tip, with all spheroids retained within their microcavities throughout the process. Supplementary Video S2: Real-time collection of HaCaT spheroids of varying sizes from six-well ULA cultures in preparation for Matrigel embedding. Under bright-field microscopy (EVOS, 4× objective), holospheres (>200 µm), merospheres (75–200 µm), and paraspheres (<75 µm) are sequentially aspirated and transferred into a pre-chilled microcentrifuge tube.
